# Molecular detection and characterization of *Anaplasma* spp. in sheep and cattle from Xinjiang, northwest China

**DOI:** 10.1186/s13071-015-0727-3

**Published:** 2015-02-19

**Authors:** Jifei Yang, Youquan Li, Zhijie Liu, Junlong Liu, Qingli Niu, Qiaoyun Ren, Ze Chen, Guiquan Guan, Jianxun Luo, Hong Yin

**Affiliations:** State Key Laboratory of Veterinary Etiological Biology, Key Laboratory of Veterinary Parasitology of Gansu Province, Lanzhou Veterinary Research Institute, Chinese Academy of Agricultural Science, Xujiaping 1, Lanzhou, Gansu 730046 P. R. China; Jiangsu Co-innovation Center for Prevention and Control of Important Animal Infectious Diseases and Zoonoses, Yangzhou, 225009 P. R. China

**Keywords:** 16S rRNA gene, *msp4* gene, Tick-borne disease

## Abstract

**Background:**

Anaplasmosis is caused by obligate intracellular bacteria in the genus *Anaplasma*. These bacterial pathogens are transmitted by ticks and impact both human and animal health. This study was conducted to determine the prevalence and molecular characterization of *Anaplasma* spp. in ruminants sampled in Xinjiang, northwest China.

**Methods:**

A survey was performed in August 2012 in rural areas of six counties in Xinjiang province. A total of 250 blood samples from ruminants were collected and tested for the presence of *Anaplasma* spp. by PCR. Positive samples were genetically characterized based on the 16S rRNA and *msp4* genes.

**Results:**

The results showed a high prevalence of *Anaplasma* spp. in ruminants, with at least three different *Anaplasma* species detected (*A. phagocytophilum*, *A. bovis* and *A. ovis*). The mean prevalence of single infection with each species was 17.6% (*A. phagocytophilum*), 4.8% (*A. bovis*) and 40.5% (*A. ovis*). Coinfection occurred in 20 (8.0%) animals. Phylogenetic analysis of the 16S rRNA gene of *A. bovis* and *A. phagocytophilum* revealed a higher degree of genetic diversity for the latter. The results for *A. ovis* showed genotypic variation among geographic regions in China. In addition, a closely related isolate to the canine pathogen *A. platys* was identified in ruminants.

**Conclusions:**

This survey revealed a high prevalence of *Anaplasma* sp. infections in sheep and cattle in the northwestern border regions of China, indicating the potential risk of transboundary disease.

## Background

The genus *Anaplasma* encompasses a group of obligate intracellular bacteria that infect a variety of cell types. These pathogens are transmitted by ticks and cause anaplasmosis in a number of animal species and humans [1]. Currently recognized species include *A. phagocytophilum* (previously recognized as *Ehrlichia equi*, *E. phagocytophila*, and the human granulocytic ehrlichiosis agent), *A. marginale*, *A. ovis*, *A. bovis* (formerly *E. bovis*) and *A. platys* (formerly *E. platys*). *A. phagocytophilum* infects neutrophils of human and animals and causes human, canine, and equine granulocytic anaplasmosis and tick fever in ruminants [1]. *A. marginale*, and *A. ovis* infect erythrocytes of domesticated and wild ruminants, while *A. bovis* causes disease in ruminants and small mammals using monocytes as their niche [2-4]. *A. platys* infects platelets and causes infectious cyclic thrombocytopenia in canines [5].

Several species of *Anaplasma* have been detected in Chinese ruminants, including *A. phagocytophilum*, *A. bovis*, *A. marginale* and *A. ovis. A. phagocytophilum* is thought to be maintained naturally in small mammal–tick cycles, with *Ixodes* ticks as vectors [1,6]. This pathogen has been previously found in sheep, goats, cattle, rabbits, rodents and ticks in several provinces of China [7-10]. *A. bovis* has been most commonly reported in cattle and buffalo from Africa, the Middle East and South America [11]. More recently, molecular evidence for the presence of *A. bovis* was reported in both goats and cattle in China [12,13]. In addition, despite little information on the occurrence of *A. marginale* and *A. ovis*, these agents are known to cause severe disease in northern China [14,15].

Xinjiang is a large, sparsely populated province in northwest China that is bordered by India, Mongolia, Russia, Kazakhstan, Kyrgyzstan, Tajikistan, Afghanistan, and Pakistan that relies heavily on sheep farming for protein. A serological survey of *A. phagocytophilum* infections has been conducted in ruminants in Xinjiang [10]. Aside from the aforementioned study, information on the prevalence of *Anaplasma* species represents a gap in knowledge. Furthermore, information is scarce on the molecular characterization of *A. phagocytophilum* and other *Anaplasma* spp. in northwest China. In the present study, we show that domestic ruminants from the northwestern border regions of China are commonly infected by distinct *Anaplasma* species. We also present molecular evidence for a potentially novel *Anaplasma* sp. closely related to *A. platys* in cattle. Our results will be useful for the risk assessment of the cross-border spread of anaplasmosis.

## Methods

### Study sites and collection of specimens

The survey was performed in August 2012 in rural areas of Kashgar, Akto, Artux, Yecheng, Pishan and Hotan counties in Xinjiang province. Sampling sites were located in the south and west of Xinjiang province, near the border with Kyrgyzstan, Tajikistan, Afghanistan and Pakistan. For each county, two to three sites were selected for sampling. Blood samples were taken from the jugular vein of 250 asymptomatic domestic ruminants (sheep and cattle, n = 125/each species) and collected in a sterile tube containing an anticoagulant (EDTA). DNA was extracted from 300 μL of blood using the Gentra Puregene DNA purification kit (Qiagen, Beijing, China) according to the manufacturer’s instructions.

### PCR reactions

The extracted DNA was examined by nested PCR for the presence of *A. phagocytophilum* and *A. bovis* 16S rRNA gene and *A. ovis* major surface protein 4 (*msp4*) gene as previously described [16,17]. The reaction was performed in an automatic thermocycler (Bio-Rad) with a total volume of 25 μL containing 2.5 μL of 10× PCR buffer (Mg^2+^ Plus), 2.0 μL of each dNTP at 2.5 mM, 1.25 U of *Taq* DNA polymerase (TaKaRa), 2.0 μL of template DNA, 1.0 μL of each primer (10 pmol), and 16.25 μL of distilled water. Genomic DNA extracted from infected animals was used as the positive control, and sterile water was used as the negative control. Cycling conditions for 16S rRNA amplification were: 4 min of denaturation at 94°C, 35 cycles at 94°C for 1 min, annealing for 1 min at 55°C, and 72°C for 1 min, with a final extension step at 72°C for 10 min. For *msp4* amplification, after an initial denaturation step of 30 s at 94°C, each cycle consisted of a denaturing step of 30 s at 94°C, an annealing for 30 s at 60°C, and an extension step of 1 min at 68°C. PCR products were visualized by UV transillumination in a 1.0% agarose gel following electrophoresis and staining with ethidium bromide.

### DNA sequencing and phylogenetic analysis

Positive PCR products were purified using the TaKaRa Agarose Gel DNA purification Kit Ver.2.0 (TaKaRa, China), ligated into pGEM-T Easy vector (Promega, USA) and transformed into *Escherichia coli* JM109 competent cells (TaKaRa, China). Two recombinant clones were selected for sequencing using BigDye Terminator Mix (Sangon, China). The obtained sequences were analyzed by a BLASTn search in GenBank or by using the Clustal W method in the MegAlign software (DNAStar, Madison, WI). A phylogenetic tree was then constructed based on the sequence distance method using the neighbor-joining (NJ) algorithm with the Kimura two-parameter model of the Mega 4.0 Software [18].

### Statistical analysis

The results were analyzed using a Chi-square test in Predictive for Analytics Software (PASW) Statistics 18. A difference was considered statistically significant at *P* < 0.05.

### Nucleotide sequence accession numbers

The GenBank accession numbers for the 16S rRNA gene sequences obtained in this study were as follows: KJ782381–KJ782387 for *A. phagocytophilum* detected in sheep, KJ782388–KJ782392 for *A. phagocythopilum* detected in cattle, and KJ782393–KJ782395 for *A. bovis* detected in cattle. The *msp4* gene sequences of *A. ovis* were assigned accession numbers KJ782396–KJ782404.

### Ethical approval

This study was approved by the Animal Ethics Committee of Lanzhou Veterinary Research Institute, Chinese Academy of Agricultural Sciences. Animals were handled in accordance with the Animal Ethics Procedures and Guidelines of the P. R. China.

## Results

Out of 250 sampled animals, 44 (17.6%) were positive for *A. phagocytophilum* (Table 1). The average positive rates were 40.5% and 28.8% for *A. ovis* and *A. phagocytophilum* in sheep (n = 125), respectively. Eighteen (14.4%) samples were simultaneously positive to *A. ovis* and *A. phagocytophilum*. For cattle (n = 125), the average positive rates were 4.8% and 6.4% for *A. bovis* and *A. phagocytophilum*, respectively. Coinfection occurred in only two (1.6%) of the sampled cattle. As shown in Table 1, three-pathogens were found in four of six study sites. In other sites, *A. ovis* was the only pathogens detected in Hotan, while *A.bovis* and *A. phagocytophilum* were found in Akto.

**Table 1:** **Detection of** ***Anaplasma*** **pathogens in sheep and cattle at various geographic sites**

**County**	**No. infected/(%)**								
	**No. tested**	**Sheep**			**No. tested**	**Cattle**			**Total**
		***A. ovis***	A. phago*	***A. ovis*** **+** ***A. phago***		***A. bovis***	***A. phago***	***A. bovis*** **+** ***A. phago***	
Yecheng	20	18 (90.0)	5 (25.0)	4 (20.0)	22	1 (5.0)	0 (0)	0 (0)	42
Akto	23	0 (0)	0 (0)	0 (0)	22	1 (4.3)	4 (17.4)	0 (0)	45
Pishan	20	10 (50.0)	1 (5.0)	1 (5.0)	21	1 (5.0)	1 (5.0)	1 (5.0)	41
Hotan	20	5 (25.0)	0 (0)	0 (0)	20	0 (0)	0 (0)	0 (0)	40
Kashgar	19	14 (73.7)	9 (47.4)	9 (47.4)	20	2 (10.5)	3 (15.8)	1 (5.3)	39
Artux	23	4 (17.4)	21 (91.3)	4 (17.4)	20	1 (4.3)	0 (0)	0 (0)	43
Total	125	51 (40.5)	36 (28.8)	18 (14.4)	125	6 (4.8)	8 (6.4)	2 (1.6)	250

*A. phago = *A. phagocytophilum*

To characterize these agents detected in sheep and cattle, positive samples representative of different hosts and geographic locations were sequenced, 25 sequences were obtained in this study. The partial 16S rRNA gene (642 and 551 bp) of *A. phagocytophilum* and *A. bovis* as well as *msp4* (869 bp) of *A. ovis* were analyzed. After BLAST and CLUSTAL W alignment, 12 PCR products of *A. phagocytophilum* chosen as representative of different hosts and geographic locations resulted in four different 16S rRNA gene sequence types. The different types of 16S rRNA sequences identified for *A. phagocytophilum* in this study are designated as 1–4. The similarity among sequence types 1–4 ranged from 97.2% to 99.5%, showcasing the genetic diversity of *A. phagocytophilum* in China. Types 1 and 2 (PS6, KS6, KS20, AKT11 and AKT4) were found to be 98.8% and 99.2% identical to strain CE18 (GenBank accession no. GQ450278) that was detected in the *Cervus elaphus* from Poland [19]. Type 3 (ATS1 and ATS15) had 98.3% identity to the HB231 strain (GenBank accession no. JN558816) derived from goat in Hubei, China [13]. Type 4 (YC38, YC29, PS19, KS9 and KS8) was 98.6% identical to strain ES34 (GenBank accession no. AB196720) that was detected in deer from Japan [16].

The 16S rRNA *A. bovis* sequences identified in cattle were 99.8 to 100% identical, despite geographic separation. Two isolates (YC7 and AKT5) were 100% identical to strain ES1019 (GenBank accession no. HQ913644), which was found in a Chinese goat. One isolate (KS2) was 99.8% identical to a strain found in Chinese cattle (GenBank accession no. FJ169957, strain name was not available). In addition, one isolate (KS6) showed high similarity (98.5%) to strains of *A. platys* (GenBank accession no. JQ894779 and AF156784). Sequence analysis of *msp4* amplicons confirmed their identity to *A. ovis*. Nine *msp4* sequences (ATS12, ATS20, HT29, HT32, KS7, KS9, PS3, YC25 and YC26) were 99.5% to 100% identical to each other and showed 99.8% to 100% identity to *A. ovis* genotype AOI identified in sheep from Italy (GenBank accession no. EU436160) [20].

**Figure 1:**
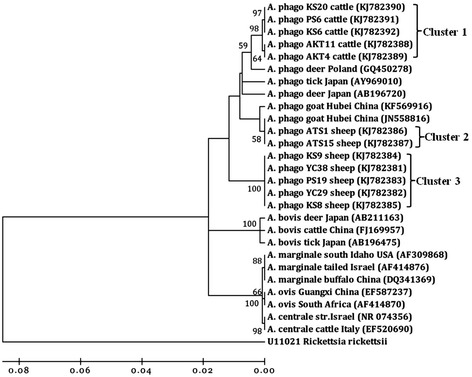
**Phylogenetic analysis of** ***A. phagocytophilum*** **(** ***A. phago*** **) based on 16S rRNA gene partial sequences.** A neighbor joining tree was constructed using the Kimura two-parameter model in Mega 4.0. An alignment of 16S rRNA sequences from position 694 to 1334 of the sequence (based on strain ES34, GenBank accession no. AB196720) was used to construct this tree. *Rickettsia rickettsii* is used as an outgroup.

**Figure 2:**
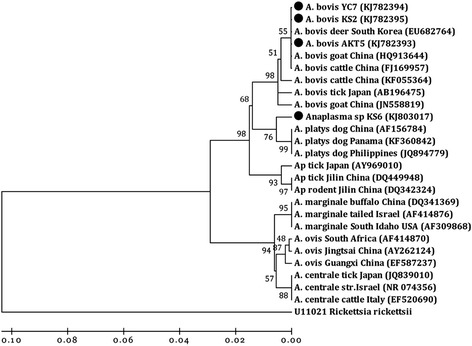
**Phylogenetic analysis of** ***A. bovis*** **based on 16S rRNA gene partial sequences.** A neighbor joining tree was constructed using the Kimura two-parameter model in Mega 4.0. An alignment of 16S rRNA sequences from position 60 to 610 of the sequence (based on strain ES1019, GenBank accession no. HQ913644) was used to construct this tree. *Rickettsia rickettsii* is used as an outgroup. ●: sequences in this study.

**Figure 3:**
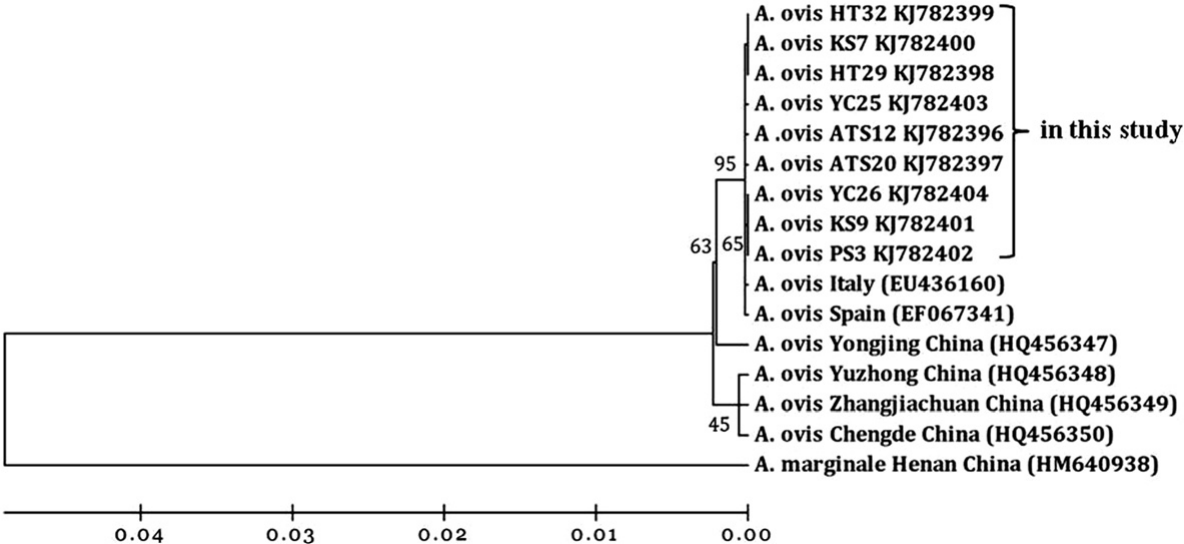
**Phylogenetic analysis of** ***A. ovis*** **strains based on the deduced amino acid sequences for the** ***msp4*** **gene.** A neighbor joining tree was constructed using the Kimura two-parameter model in Mega 4.0. An alignment of full length Msp4 sequences was used to construct this tree.

Phylogenetic analyses indicated that the 16S rRNA sequence types from *A. phagocytophilum* formed three main clusters, implying geographic and host segregation of strains in China (Figure 1). Cluster one displayed a close relationship with the sequence amplified from deer found in Poland (GenBank accession no. GQ450278). Cluster two showed a close relationship with the sequence amplified from a goat in central China (GenBank accession no. KF569916 and JN558816). Cluster three clustered independently from all known *A. phagocytophilum* sequences. Three isolates of *A. bovis* were classified into a cluster together with isolates from Chinese goat and cattle (GenBank accession no. HQ913644 and FJ169957) as well as South Korean deer (GenBank accession no. AB682764) (Figure 2). No geographic segregation of *A. bovis* isolates was observed in this study. One sequences derived from cattle showed a close relationship with *A. platys* and grouped in a separate clade with isolate Gzh981 from a Chinese dog (GenBank accession no. AF156784) (Figure 2), indicating that cattle are an alternative host of *A. platys* in China. Nine isolates of *A. ovis* were closely related to *A. ovis* genotype AOI reported in Italy (GenBank accession no. EU436160) and separated from isolates reported in China (GenBank accession no. HQ456347-HQ456350) (Figure 3).

## Discussion

In this study, we performed a molecular survey of *Anaplasma* pathogens of human and veterinary interest in northwest China. Our findings revealed that not only are *A. phagocytophilum*, *A. bovis* and *A. ovis* present in animals from different study sites, but an *A. platys*-like pathogen was also present.

*A. phagocytophilum* is known as an emerging human pathogen of public health relevance [21]. In addition to humans, many domestic animals, such as dogs, cats, horses, sheep, goats, and cattle, can become infected with *A. phagocytophilum* and show clinical signs with high fever, depression, anorexia and weight loss [22]. Previous reports revealed that the infection rates for *A. phagocytophilum* were variable in different hosts and geographic locations in China. Several molecular surveys have shown that *A. phagocytophilum* infection rates of: 5.7% (8/35), 13.0% (6/46), 14.5% (10/69), 38.5% (35/91) in goats from Jilin, Henan, Hubei and Gansu; 7.1% (5/70) and 42.9% (21/49) in sheep from Jilin and Gansu; 6.8% (38/557) in rodents from Heilongjiang, Jilin and Zhejiang; and 3.42% (83/2429) in ticks from different sites near the China-Russia border [8,13,23,24]. In the present study, the average positive rates of *A. phagocytophilum* were 28.8% and 6.4% in sheep and cattle, respectively. The positive rate was significantly higher in sheep than in cattle (*P* < 0.001). However, it is hard to conclude that sheep were a more suitable host for *A. phagocytophilum* in our study sites as the difference in infection rates could be explained by the known tick vectors that transmit *A. phagocytophilum. A. phagocytophilum* is usually associated with ticks of the genus *Ixodes* [25,26]. However, because of its capacity for transstadial and transovarial transmission, *Dermacentor albipictus* may be another vector for *A. phagocytophilum* [27]. Moreover, *A. phagocytophilum* DNA has been detected in *H. concinna*, *H. longicornis*, *H. qinghaiensis*, *I. persulcatus* and *Dermacentor silvarum* in China [23,24,26], indicating that numerous tick species may maintain or be involved in the transmission of *A. phagocytophilum*. As a number of tick species are considered to be host specific [28], we assume that the sheep were infested with tick vectors that preferentially feed on sheep as compared to cattle, and that this resulted in a higher prevalence of *A. phagocytophilum* in sheep. These results indicated that both sheep and cattle are part of the natural maintenance cycle of *A. phagocytophilum. A. phagocytophilum* appears to exhibit ecotypes with different host ranges and zoonotic potential [29], It is unclear at this stage how the Chinese genotypes would segregate into ecotypes, however, in a European study genotypes that infected cattle were in the same ecotype as those that infected humans [29]. Clearly, the finding of cattle infected with *A. phagocytophilum* warrants further investigation.

The *A. phagocytophilum* 16S rRNA gene sequences identified herein were analyzed together with sequences reported previously for the characterization of the genetic diversity of *A. phagocytophilum* strains in comparison with other *Anaplasma* spp. Four different 16S rRNA genotypes of *A. phagocytophilum* were identified in this study, indicating that *A. phagocytophilum* is genetically diverse within China. Similar results have been reported in goats from central and southern China [13]. Phylogenetic analyses revealed these genotypes formed three main clusters. Interestingly, the genotypes 1 and 2 (99.5% similarity) derived from cattle were classified into one cluster and separated from type 3 and 4 derived from sheep. This result suggested that *A. phagocytophilum* genotypes may vary between sheep and cattle. Several studies reported that *A. phagocytophilum* strains differ in host infectivity [29-31]. In this study, cluster 1 (genotypes 1 and 2) were more likely to infect cattle; while cluster 2 (genotype 3) and 3 (genotype 4) were more likely to infect sheep. In addition, cluster 3 was in a divergent cluster from the other *A. phagocytophilum* sequences, implying that it is genetically distinct from the known *A. phagocytophilum* strains described in China (Figure 1).

*A. bovis* has a wide host range, encompassing both domestic and wild mammals. Susceptible species include cattle, goats, dogs, cats and deer [11,13,16,32,33]. Investigation of *A. bovis* infections in cattle showed a prevalence that ranged between 0 and 10.5%, with an average of 4.8%, which was significantly lower than the prevalence in cattle (53.3%) in Japan [11]. In recent years, molecular evidence for *A. bovis* infection in goats showed a higher prevalence of 49.6% in central and southern China [13], and in sheep and goats in southwestern China [12]. Considering this, we sought to determine whether the *A. bovis* was present in sheep at our study sites. Unfortunately, *A. bovis* DNA was not detected in sheep samples in this study. Sequences analysis revealed that *A. bovis* amplified from our sampled cattle were grouped into one cluster and had high identity with isolates from Chinese goat and cattle. According to our results, and in contrast to *A. phagocytophilum*, no host segregation or geographical isolation was observed among *A. bovis* strains in China. Surprisingly, despite the capacity of *A. bovis* to infect multiple hosts and its wide distribution, *A. bovis* 16S rRNA gene sequences were less genetically diverse compared with *A. phagocytophilum* (Figure 2).

*A. platys* shows unique tropism for dog platelets, being the etiological agent of the infectious canine cyclic thrombocytopenia [4]. Interestingly, an *A. platys*-like pathogen was found in cattle from Kashgar county in this study. This isolate (KS6, KJ803017) was closely related to *A. platys* and is a sister taxon to *A. platys* isolates in dogs from China, Panama and the Philippines (Figure 2). This is not the first report for *A. platys*-like pathogen detected in ruminants. In previous studies, *A. platys*-like organisms have been reported in goats from central and southern China [13], and in sheep, goats and cattle with a high prevalence (55.6%, 55/99) from the Island of Sardinia, Italy [4]. These results suggest that ruminants are a likely alternative host for *A. platys*.

The average infection rate of *A. ovis* (40.5%) in sheep was slightly lower than that in goats (with an average of 46.6%) from central and southern China (*P* > 0.05), indicating wide distribution and enzootic stability [13]. Phylogenetic analysis of *msp4* sequences revealed one *A. ovis msp4* genotype in sheep that was closely related to *A. ovis* genotype AOI from Italy (GenBank accession no. EU436160) [20]. A previous study identified six *A. ovis msp4* genotypes in sheep and goats from China [13]. However, the isolates in Xinjiang were phylogenetically separated from all of them, indicating genotypic variation among geographic regions. Considering *Anaplasma* major surface proteins (MSPs) are likely to evolve more rapidly than other genes because they are subjected to selective pressures exerted by host immune systems [17], the variation of *msp4* sequences observed in sheep and goats was low (Figure 3).

Finally, coinfection occurred in 20 (8.0%) of the sampled animals. The coinfection rate (18/125, 14.4%) of *A. phagocytophilum* and *A. ovis* in sheep was significantly higher than the coinfection rate (2/125, 1.6%) of *A. phagocytophilum* and *A. bovis* in cattle (*P* < 0.01). In addition, one sample from cattle (KS6) was simultaneously positive for *A. phagocytophilum* and an *A. platys*-like organism. Although *Anaplasma* species show different preferential host and cell tropism [1,3,4], coinfection increases the potential difficulty in diagnosis of *Anaplasma* infection. Furthermore, investigation of potential tick vectors will be useful for understanding the life cycle and promoting a comprehensive strategy to both prevent and control these pathogens.

## Conclusions

Infections with *A. phagocytophilum*, *A. bovis* and *A. ovis* bacteria are endemic in ruminants from northwest China. Our survey revealed a high prevalence of *Anaplasma* sp. infections in sheep and cattle in the northwestern border regions of China, indicating a potential risk for transboundary disease.
